# Incidence of venous thromboembolism in japanese trauma population

**DOI:** 10.1186/2197-425X-3-S1-A378

**Published:** 2015-10-01

**Authors:** R Miki, S Kikuta, T Koga, S Kai, A Inoue, T Kawase, S Ishihara, S Nakayama

**Affiliations:** Emergency Medicine and Critical Care, Hyogo Emergency Medical Center, Kobe, Japan

## Introduction

There have been several manuscripts reporting Asians having lower risk for developing venous thromboembolism (VTE) in the medical population. However, incidence of VTE in the Asian trauma population is not fully known.

## Objectives

To investigate the incidence of deep venous thrombosis (DVT) and massive or submassive pulmonary embolism (PE) in Japanese trauma population.

## Methods

This single center observational study was conducted at tertiary emergency and critical care center in Japan. From April 2009 to March 2015, all trauma patients admitted to intensive care unit (ICU) were enrolled in this study. To investigate the incidence of DVT, all patients were risk stratified according to VTE risk assessment checklist on admission and adequate thromboprophylaxis was performed. Patients were screened for DVT by lower extremity duplex ultrasonography (US) six-days after admission and the incidence of DVT was assessed. DVT in popliteal vein or above was defined proximal and others were defined distal. To investigate the incidence of PE, all of the computed tomography (CT) reports were searched retrospectively by keyword “PE” or “VTE” during the study period including autopsy imaging. Massive and submassive PE was defined by both presence of thrombi in the main pulmonary artery and right ventricular diameter greater than that of left ventricle by contrast enhanced CT exam, suggesting of right ventricular pressure load. Patients who had PE by contrast enhanced CT either by coincidence or due to clinical symptom were identified.

## Results

During the six-year study period, 2,618 trauma patients were enrolled. Mean age was 48.7 years and 1,885 patients (72%) were male. Blunt injury accounted for 2,339 patients (89%), and mean injury severity score was 20. Blood products were transfused in 572 patients (22%) and 1,144 (44%) required operation or interventional radiology within 24 hours of arrival. In-hospital mortality was 13%. By using duplex US, 916 patients were screened for DVT. Of those 916 patients, 29 patients (3%) developed proximal DVT and 202 patients (22%) developed distal DVT during ICU stay. Among 2,618 trauma patients, 2 patients (0.2%) developed massive or submassive PE during ICU stay, of which one died due to PE. There were 10 cases (0.4%) of non-massive or non-submassive PE identified by contrast enhanced CT as well.

## Conclusions

The incidence of proximal and distal DVT in Japanese trauma population admitted to ICU was 3% and 22% respectively. The incidence of massive or submassive PE was 0.2%. A larger study is warranted to reveal an accurate risk of VTE and determine optimal anti-coagulation measure in Asian trauma population, considering low incidence of VTE in this study.

## References

[1] White RH (2003). The Epidemiology of Venous Thromboembolism. Circulation.

[2] Barrera LM (2013). Thromboprophylaxis for trauma patients. Cochrane Database Syst Rev.

